# Supplementary material for: Less Severe Sepsis in Cecal Ligation and Puncture Models with and without Lipopolysaccharide in Mice with Conditional Ezh2-Deleted Macrophages (LysM-Cre System)
Source: Int J Mol Sci. 2023 May 10;24(10):8517. doi: 10.3390/ijms24108517 (PMC10218384; doi:10.3390/ijms24108517)
Supplement: Supplementary file 1 [file ijms-24-08517-s001.zip › ijms-2362927-supplementary.pdf]

Supplementary Materials

Less Severe Sepsis in Cecal Ligation and Puncture Models with and without Lipopolysaccharide in Mice with Conditional *Ezh2*-Deleted Macrophages (LysM-Cre System)

Supplement Table S1. The unique proteins from cell lysate (proteome) that presented only in LPS-activated Ezh2 null macrophages

Pasted#	Symbol	Chr*	Position (Mbp)	Description
ADK	Adk	14	21.10264	adenosine kinase [Source:MGI Symbol;Acc:MGI:87930]
AIF1	Aif1	17	35.38997	allograft inflammatory factor 1 [Source:MGI Symbol;Acc:MGI:1343098]
ANKRD13A	Ankrd13a	5	114.9127	ankyrin repeat domain 13a [Source:MGI Symbol;Acc:MGI:1915670]
CCL4	Ccl4	11	83.55341	chemokine (C-C motif) ligand 4 [Source:MGI Symbol;Acc:MGI:98261]
CD14	Cd14	18	36.85812	CD14 antigen [Source:MGI Symbol;Acc:MGI:88318]
CD40	Cd40	2	164.8975	CD40 antigen [Source:MGI Symbol;Acc:MGI:88336]
CMPK2	Cmpk2	12	26.5192	cytidine monophosphate (UMP-CMP) kinase 2, mitochondrial [Source:MGI Symbol;Acc:MGI:99830]
DTX3L	Dtx3l	16	35.74688	deltex 3-like, E3 ubiquitin ligase [Source:MGI Symbol;Acc:MGI:2656973]
F13A1	F13a1	13	37.05115	coagulation factor XIII, A1 subunit [Source:MGI Symbol;Acc:MGI:1921395]
HCK	Hck	2	152.9504	hemopoietic cell kinase [Source:MGI Symbol;Acc:MGI:96052]
MAPRE1	Mapre1	2	153.5832	microtubule-associated protein, RP/EB family, member 1 [Source:MGI Symbol;Acc:MGI:891995]
MARCKSL1	Marcksl1	4	129.4074	MARCKS-like 1 [Source:MGI Symbol;Acc:MGI:97143]
NPL	Npl	1	153.3788	N-acetylneuraminate pyruvate lyase [Source:MGI Symbol;Acc:MGI:1921341]
NRP2	Nrp2	1	62.74244	neuropilin 2 [Source:MGI Symbol;Acc:MGI:1100492]
PARP14	Parp14	16	35.65324	poly (ADP-ribose) polymerase family, member 14 [Source:MGI Symbol;Acc:MGI:1919489]
PCBD2	Pcbd2	13	55.87518	pterin 4 alpha carbinolamine dehydratase/dimerization cofactor of hepatocyte nuclear factor 1 alpha (TCF1) 2 [Source:MGI Symbol;Acc:MGI:1919812]
PIN1	Pin1	9	20.56339	protein (peptidyl-prolyl cis/trans isomerase) NIMA-interacting 1 [Source:MGI Symbol;Acc:MGI:1346036]
PKN1	Pkn1	8	84.39317	protein kinase N1 [Source:MGI Symbol;Acc:MGI:108022]
PTGS2	Ptgs2	1	149.9758	prostaglandin-endoperoxide synthase 2 [Source:MGI Symbol;Acc:MGI:97798]
RAP2C	Rap2c		50.09279	RAP2C, member of RAS oncogene family [Source:MGI Symbol;Acc:MGI:1919315]
RPL23	Rpl23	11	97.66835	ribosomal protein L23 [Source:MGI Symbol;Acc:MGI:1929455]
RSAD2	Rsad2	12	26.49275	radical S-adenosyl methionine domain containing 2 [Source:MGI Symbol;Acc:MGI:1929628]
SAFB	Safb	17	56.89183	scaffold attachment factor B [Source:MGI Symbol;Acc:MGI:2146974]
SLFN5	Slfn5	11	82.84218	schlafen 5 [Source:MGI Symbol;Acc:MGI:1329004]
SRA1	Sra1	18	36.79973	steroid receptor RNA activator 1 [Source:MGI Symbol;Acc:MGI:1344414]
TIMM10	Timm10	2	84.65734	translocase of inner mitochondrial membrane 10 [Source:MGI Symbol;Acc:MGI:1353429]

TNFAIP2	Tnfaip2	12	111.4089	tumor necrosis factor, alpha-induced protein 2 [Source:MGI Symbol;Acc:MGI:104960]
TREX1	Trex1	9	108.887	three prime repair exonuclease 1 [Source:MGI Symbol;Acc:MGI:1328317]
TRIM14	Trim14	4	46.49378	tripartite motif-containing 14 [Source:MGI Symbol;Acc:MGI:1921985]
UBA2	Uba2	7	33.84011	ubiquitin-like modifier activating enzyme 2 [Source:MGI Symbol;Acc:MGI:1858313]
ZNF706	Zfp706	15	36.99727	zinc finger protein 706 [Source:MGI Symbol;Acc:MGI:1915286]

* Chr, Chromosome

Supplement Table S2. The unique proteins from cell lysate (proteome) that presented only in Ezh2 null macrophages with LPS tolerance

Pasted	Symbol	Chr	Position (Mbp)	Description
NRP2	Nrp2	1	62.74244	neuropilin 2 [Source:MGI Symbol;Acc:MGI:1100492]
RALB	Ralb	1	119.398	v-ral simian leukemia viral oncogene B [Source:MGI Symbol;Acc:MGI:1927244]
RAB3GAP1	Rab3gap1	1	127.7965	RAB3 GTPase activating protein subunit 1 [Source:MGI Symbol;Acc:MGI:2445001]
NPL	Npl	1	153.3788	N-acetylneuraminate pyruvate lyase [Source:MGI Symbol;Acc:MGI:1921341]
SERPINC1	Serpinc1	1	160.8062	serine (or cysteine) peptidase inhibitor, clade C (antithrombin), member 1 [Source:MGI Symbol;Acc:MGI:88095]
DCAF8	Dcaf8	1	171.9757	DDB1 and CUL4 associated factor 8 [Source:MGI Symbol;Acc:MGI:91860]
NVL	Nvl	1	180.9147	nuclear VCP-like [Source:MGI Symbol;Acc:MGI:1914709]
NUDT5	Nudt5	2	5.84983	nudix (nucleoside diphosphate linked moiety X)-type motif 5 [Source:MGI Symbol;Acc:MGI:1858232]
RSU1	Rsu1	2	13.08163	Ras suppressor protein 1 [Source:MGI Symbol;Acc:MGI:103040]
YME1L1	Yme1l1	2	23.04638	YME1-like 1 (S. cerevisiae) [Source:MGI Symbol;Acc:MGI:1351651]
HNMT	Hnmt	2	23.89292	histamine N-methyltransferase [Source:MGI Symbol;Acc:MGI:2153181]
MTCH2	Mtch2	2	90.6775	mitochondrial carrier 2 [Source:MGI Symbol;Acc:MGI:1929260]
EMC4	Emc4	2	112.1934	ER membrane protein complex subunit 4 [Source:MGI Symbol;Acc:MGI:1915282]
HYPK	Hypk	2	121.2838	huntingtin interacting protein K [Source:MGI Symbol;Acc:MGI:1914943]
NFS1	Nfs1	2	155.9656	nitrogen fixation gene 1 (S. cerevisiae) [Source:MGI Symbol;Acc:MGI:1316706]
TOP1	Top1	2	160.4878	topoisomerase (DNA) I [Source:MGI Symbol;Acc:MGI:98788]
MRPS28	Mrps28	3	8.867206	mitochondrial ribosomal protein S28 [Source:MGI Symbol;Acc:MGI:1913480]
NHLRC3	Nhlrc3	3	53.356	NHL repeat containing 3 [Source:MGI Symbol;Acc:MGI:2444520]
RPS27	Rps27	3	90.11983	ribosomal protein S27 [Source:MGI Symbol;Acc:MGI:1888676]
RBM8A	Rbm8a	3	96.53725	RNA binding motif protein 8a [Source:MGI Symbol;Acc:MGI:1913129]
NRAS	Nras	3	102.9656	neuroblastoma ras oncogene [Source:MGI Symbol;Acc:MGI:97376]
PPA2	Ppa2	3	133.0159	pyrophosphatase (inorganic) 2 [Source:MGI Symbol;Acc:MGI:1922026]

CRYZ	Cryz	3	154.3023	crystallin, zeta [Source:MGI Symbol;Acc:MGI:88527]
NDUFB6	Ndufb6	4	40.27059	NADH:ubiquinone oxidoreductase subunit B6 [Source:MGI Symbol;Acc:MGI:2684983]
STX17	Stx17	4	48.12492	syntaxin 17 [Source:MGI Symbol;Acc:MGI:1914977]
SMC2	Smc2	4	52.43924	structural maintenance of chromosomes 2 [Source:MGI Symbol;Acc:MGI:106067]
COA7	Coa7	4	108.1853	cytochrome c oxidase assembly factor 7 [Source:MGI Symbol;Acc:MGI:1917143]
RRAGC	Rragc	4	123.8112	Ras-related GTP binding C [Source:MGI Symbol;Acc:MGI:1858751]
ALDH4A1	Aldh4a1	4	139.3502	aldehyde dehydrogenase 4 family, member A1 [Source:MGI Symbol;Acc:MGI:2443883]
FBLIM1	Fblim1	4	141.3034	filamin binding LIM protein 1 [Source:MGI Symbol;Acc:MGI:1921452]
DNAJC16	Dnajc16	4	141.4875	DnaJ heat shock protein family (Hsp40) member C16 [Source:MGI Symbol;Acc:MGI:2442146]
ATAD3	Atad3a	4	155.8251	ATPase family, AAA domain containing 3A [Source:MGI Symbol;Acc:MGI:1919214]
MLEC	Mlec	5	115.281	malectin [Source:MGI Symbol;Acc:MGI:1924015]
DDX54	Ddx54	5	120.7508	DEAD box helicase 54 [Source:MGI Symbol;Acc:MGI:1919240]
P2RX4	P2rx4	5	122.8456	purinergic receptor P2X, ligand-gated ion channel 4 [Source:MGI Symbol;Acc:MGI:1338859]
TMEM120A	Tmem120a	5	135.7643	transmembrane protein 120A [Source:MGI Symbol;Acc:MGI:2686991]
NDUFA5	Ndufa5	6	24.51867	NADH:ubiquinone oxidoreductase subunit A5 [Source:MGI Symbol;Acc:MGI:1915452]
IRF5	Irf5	6	29.52662	interferon regulatory factor 5 [Source:MGI Symbol;Acc:MGI:1350924]
CHCHD6	Chchd6	6	89.36013	coiled-coil-helix-coiled-coil-helix domain containing 6 [Source:MGI Symbol;Acc:MGI:1913348]
ALOX5	Alox5	6	116.387	arachidonate 5-lipoxygenase [Source:MGI Symbol;Acc:MGI:87999]
UBE2M	Ube2m	7	12.76905	ubiquitin-conjugating enzyme E2M [Source:MGI Symbol;Acc:MGI:108278]
AP2S1	Ap2s1	7	16.47234	adaptor-related protein complex 2, sigma 1 subunit [Source:MGI Symbol;Acc:MGI:2141861]
ECH1	Ech1	7	28.52464	enoyl coenzyme A hydratase 1, peroxisomal [Source:MGI Symbol;Acc:MGI:1858208]
EMC10	Emc10	7	44.13936	ER membrane protein complex subunit 10 [Source:MGI Symbol;Acc:MGI:1916933]
AKT1S1	Akt1s1	7	44.49842	AKT1 substrate 1 (proline-rich) [Source:MGI Symbol;Acc:MGI:1914855]
SNRPA1	Snrpa1	7	65.70875	small nuclear ribonucleoprotein polypeptide A' [Source:MGI Symbol;Acc:MGI:1916231]
THUMPD1	Thumpd1	7	119.3143	THUMP domain containing 1 [Source:MGI Symbol;Acc:MGI:2444479]
IFITM3	Ifitm3	7	140.5895	interferon induced transmembrane protein 3 [Source:MGI Symbol;Acc:MGI:1913391]
NAXD	Naxd	8	11.54751	NAD(P)HX dehydratase [Source:MGI Symbol;Acc:MGI:1913353]
PPP2CB	Ppp2cb	8	34.08965	protein phosphatase 2 (formerly 2A), catalytic subunit, beta isoform [Source:MGI Symbol;Acc:MGI:1321161]

DCTN6	Dctn6	8	34.55757	dynactin 6 [Source:MGI Symbol;Acc:MGI:1343154]
SNTB2	Sntb2	8	107.6624	syntrophin, basic 2 [Source:MGI Symbol;Acc:MGI:101771]
NRP1	Nrp1	8	129.0851	neuropilin 1 [Source:MGI Symbol;Acc:MGI:106206]
DNMT1	Dnmt1	9	20.81851	DNA methyltransferase (cytosine-5) 1 [Source:MGI Symbol;Acc:MGI:94912]
ATP5MG	Atp5l	9	44.82386	ATP synthase, H+ transporting, mitochondrial F0 complex, subunit G [Source:MGI Symbol;Acc:MGI:1351597]
ADPGK	Adpgk	9	59.19884	ADP-dependent glucokinase [Source:MGI Symbol;Acc:MGI:1919391]
UACA	Uaca	9	60.70182	uveal autoantigen with coiled-coil domains and ankyrin repeats [Source:MGI Symbol;Acc:MGI:1919815]
ADAM10	Adam10	9	70.58628	a disintegrin and metallopeptidase domain 10 [Source:MGI Symbol;Acc:MGI:109548]
SLC17A5	Slc17a5	9	78.44377	solute carrier family 17 (anion/sugar transporter), member 5 [Source:MGI Symbol;Acc:MGI:1924105]
MTHFD1L	Mthfd1l	10	3.923118	methylenetetrahydrofolate dehydrogenase (NADP+ dependent) 1-like [Source:MGI Symbol;Acc:MGI:1924836]
NUP43	Nup43	10	7.543267	nucleoporin 43 [Source:MGI Symbol;Acc:MGI:1917162]
ARG1	Arg1	10	24.79112	arginase, liver [Source:MGI Symbol;Acc:MGI:88070]
ARL1	Arl1	10	88.56672	ADP-ribosylation factor-like 1 [Source:MGI Symbol;Acc:MGI:99436]
BLOC1S1	Bloc1s1	10	128.7538	biogenesis of lysosomal organelles complex-1, subunit 1 [Source:MGI Symbol;Acc:MGI:1195276]
PNPT1	Pnpt1	11	29.08074	polyribonucleotide nucleotidyltransferase 1 [Source:MGI Symbol;Acc:MGI:1918951]
MAP2K3	Map2k3	11	60.82286	mitogen-activated protein kinase kinase 3 [Source:MGI Symbol;Acc:MGI:1346868]
PLSCR3	Plscr3	11	69.7372	phospholipid scramblase 3 [Source:MGI Symbol;Acc:MGI:1917560]
NUP88	Nup88	11	70.83388	nucleoporin 88 [Source:MGI Symbol;Acc:MGI:104900]
CPD	Cpd	11	76.66925	carboxypeptidase D [Source:MGI Symbol;Acc:MGI:107265]
SDF2	Sdf2	11	78.13657	stromal cell derived factor 2 [Source:MGI Symbol;Acc:MGI:108019]
ACSF2	Acsf2	11	94.44793	acyl-CoA synthetase family member 2 [Source:MGI Symbol;Acc:MGI:2388287]
FDXR	Fdxr	11	115.1589	ferredoxin reductase [Source:MGI Symbol;Acc:MGI:104724]
SUMO2	Sumo2	11	115.4139	small ubiquitin-like modifier 2 [Source:MGI Symbol;Acc:MGI:2158813]
PPM1A	Ppm1a	12	72.80423	protein phosphatase 1A, magnesium dependent, alpha isoform [Source:MGI Symbol;Acc:MGI:99878]
HSPA2	Hspa2	12	76.45095	heat shock protein 2 [Source:MGI Symbol;Acc:MGI:96243]
ALDH6A1	Aldh6a1	12	84.47749	aldehyde dehydrogenase family 6, subfamily A1 [Source:MGI Symbol;Acc:MGI:1915077]
GLRX5	Glrx5	12	104.9989	glutaredoxin 5 [Source:MGI Symbol;Acc:MGI:1920296]
NDUFS4	Ndufs4	13	114.4243	NADH:ubiquinone oxidoreductase core subunit S4 [Source:MGI Symbol;Acc:MGI:1343135]
RPL15	Rpl15	14	4.198305	ribosomal protein L15 [Source:MGI Symbol;Acc:MGI:1913730]
GNG2	Gng2	14	19.92263	guanine nucleotide binding protein (G protein), gamma 2 [Source:MGI Symbol;Acc:MGI:102705]

CTSG	Ctsg	14	56.33734	cathepsin G [Source:MGI Symbol;Acc:MGI:88563]
ZNF622	Zfp622	15	25.98445	zinc finger protein 622 [Source:MGI Symbol;Acc:MGI:1289282]
RIDA	Rida	15	34.48417	reactive intermediate imine deaminase A homolog [Source:MGI Symbol;Acc:MGI:1095401]
ZNF706	Zfp706	15	36.99727	zinc finger protein 706 [Source:MGI Symbol;Acc:MGI:1915286]
NMRAL1	Nmral1	16	4.527923	NmrA-like family domain containing 1 [Source:MGI Symbol;Acc:MGI:1915074]
LRCH3	Lrch3	16	32.73447	leucine-rich repeats and calponin homology (CH) domain containing 3 [Source:MGI Symbol;Acc:MGI:1917394]
MRPL39	Mrpl39	16	84.51446	mitochondrial ribosomal protein L39 [Source:MGI Symbol;Acc:MGI:1351620]
CUTA	Cuta	17	27.15279	cutA divalent cation tolerance homolog [Source:MGI Symbol;Acc:MGI:1914925]
NDUFA7	Ndufa7	17	34.04355	NADH:ubiquinone oxidoreductase subunit A7 [Source:MGI Symbol;Acc:MGI:1913666]
HSD17B8	H2-Ke6	17	34.24501	H2-K region expressed gene 6 [Source:MGI Symbol;Acc:MGI:95911]
DNPH1	Dnph1	17	46.80764	2′-deoxynucleoside 5′-phosphate N-hydrolase 1 [Source:MGI Symbol;Acc:MGI:3039376]
MRPL2	Mrpl2	17	46.95716	mitochondrial ribosomal protein L2 [Source:MGI Symbol;Acc:MGI:1351622]
HEATR5B	Heatr5b	17	79.06033	HEAT repeat containing 5B [Source:MGI Symbol;Acc:MGI:2444098]
DMXL1	Dmxl1	18	49.96574	Dmx-like 1 [Source:MGI Symbol;Acc:MGI:2443926]
TMX3	Tmx3	18	90.52828	thioredoxin-related transmembrane protein 3 [Source:MGI Symbol;Acc:MGI:2442418]
NDUFV1	Ndufv1	19	4.057384	NADH:ubiquinone oxidoreductase core subunit V1 [Source:MGI Symbol;Acc:MGI:107851]
CCS	Ccs	19	4.875394	copper chaperone for superoxide dismutase [Source:MGI Symbol;Acc:MGI:1333783]
CYCS	Gm10053	19	24.85305	predicted gene 10053 [Source:MGI Symbol;Acc:MGI:3704493]
AK3	Ak3	19	28.99823	adenylate kinase 3 [Source:MGI Symbol;Acc:MGI:1860835]
RAP2C	Rap2c		50.09279	RAP2C, member of RAS oncogene family [Source:MGI Symbol;Acc:MGI:1919315]
IDH3G	Idh3g		72.82257	isocitrate dehydrogenase 3 (NAD+), gamma [Source:MGI Symbol;Acc:MGI:1099463]
RPS6KA3	Rps6ka3		157.9933	ribosomal protein S6 kinase polypeptide 3 [Source:MGI Symbol;Acc:MGI:104557]

*Chr, Chromosome

Supplement Table S3. The unique proteins from the supernatant (secretome) that presented only in LPS-activated Ezh2 null macrophages

Pasted	Symbol	Chr*	Position (Mbp)	Description
HSPD1	Hspd1	1	55.11699	heat shock protein 1 (chaperonin) [Source:MGI Symbol;Acc:MGI:96242]
IDH1	Idh1	1	65.19778	isocitrate dehydrogenase 1 (NADP+), soluble [Source:MGI Symbol;Acc:MGI:96413]
PRDX6	Prdx6	1	161.0677	peroxiredoxin 6 [Source:MGI Symbol;Acc:MGI:894320]
VIM	Vim	2	13.57874	vimentin [Source:MGI Symbol;Acc:MGI:98932]
UAP1L1	Uap1l1	2	25.2499	UDP-N-acteylglucosamine pyrophosphorylase 1-like 1 [Source:MGI Symbol;Acc:MGI:2443318]
GSN	Gsn	2	35.14639	gelsolin [Source:MGI Symbol;Acc:MGI:95851]
CST3	Cst3	2	148.7136	cystatin C [Source:MGI Symbol;Acc:MGI:102519]
YWHAB	Ywhab	2	163.8369	tyrosine 3-monooxygenase/tryptophan 5-monooxygenase activation protein, beta polypeptide [Source:MGI Symbol;Acc:MGI:1891917]
PLTP	Pltp	2	164.6814	phospholipid transfer protein [Source:MGI Symbol;Acc:MGI:103151]
FABP5	Fabp5	3	10.07761	fatty acid binding protein 5, epidermal [Source:MGI Symbol;Acc:MGI:101790]
LMNA	Lmna	3	88.38745	lamin A [Source:MGI Symbol;Acc:MGI:96794]
S100A4	S100a4	3	90.51108	S100 calcium binding protein A4 [Source:MGI Symbol;Acc:MGI:1330282]
S100A6	S100a6	3	90.52019	S100 calcium binding protein A6 (calcyclin) [Source:MGI Symbol;Acc:MGI:1339467]
CTSS	Ctss	3	95.4341	cathepsin S [Source:MGI Symbol;Acc:MGI:107341]
CAPZA1	Capza1	3	104.7301	capping protein (actin filament) muscle Z-line, alpha 1 [Source:MGI Symbol;Acc:MGI:106227]
AKR1A1	Akr1a1	4	116.4937	aldo-keto reductase family 1, member A1 (aldehyde reductase) [Source:MGI Symbol;Acc:MGI:1929955]
PRDX1	Prdx1	4	116.5427	peroxiredoxin 1 [Source:MGI Symbol;Acc:MGI:99523]
MARCKSL1	Marcksl1	4	129.4074	MARCKS-like 1 [Source:MGI Symbol;Acc:MGI:97143]
SH3BGRL3	Sh3bgrl3	4	133.8547	SH3 domain binding glutamic acid-rich protein-like 3 [Source:MGI Symbol;Acc:MGI:1920973]
C1QB	C1qb	4	136.6074	complement component 1, q subcomponent, beta polypeptide [Source:MGI Symbol;Acc:MGI:88224]
C1QC	C1qc	4	136.6171	complement component 1, q subcomponent, C chain [Source:MGI Symbol;Acc:MGI:88225]
C1QA	C1qa	4	136.6232	complement component 1, q subcomponent, alpha polypeptide [Source:MGI Symbol;Acc:MGI:88223]
ENO1	Eno1	4	150.3212	enolase 1, alpha non-neuron [Source:MGI Symbol;Acc:MGI:95393]
PARK7	Park7	4	150.9816	Parkinson disease (autosomal recessive, early onset) 7 [Source:MGI Symbol;Acc:MGI:2135637]
WDR1	Wdr1	5	38.68416	WD repeat domain 1 [Source:MGI Symbol;Acc:MGI:1337100]
CXCL2	Cxcl2	5	91.05173	chemokine (C-X-C motif) ligand 2 [Source:MGI Symbol;Acc:MGI:1340094]
CXCL10	Cxcl10	5	92.4945	chemokine (C-X-C motif) ligand 10 [Source:MGI Symbol;Acc:MGI:1352450]
RAN	Ran	5	129.0971	RAN, member RAS oncogene family [Source:MGI Symbol;Acc:MGI:1333112]
GUSB	Gusb	5	130.0179	glucuronidase, beta [Source:MGI Symbol;Acc:MGI:95872]

MDH2	Mdh2	5	135.8073	malate dehydrogenase 2, NAD (mitochondrial) [Source:MGI Symbol;Acc:MGI:97050]
ACTB	Actb	5	142.8889	actin, beta [Source:MGI Symbol;Acc:MGI:87904]
GPNMB	Gpnmb	6	49.01348	glycoprotein (transmembrane) nmb [Source:MGI Symbol;Acc:MGI:1934765]
CAPG	Capg	6	72.52137	capping protein (actin filament), gelsolin-like [Source:MGI Symbol;Acc:MGI:1098259]
TPI1	Tpi1	6	124.7875	triosephosphate isomerase 1 [Source:MGI Symbol;Acc:MGI:98797]
GAPDH	Gapdh	6	125.1387	glyceraldehyde-3-phosphate dehydrogenase [Source:MGI Symbol;Acc:MGI:95640]
GPI	Gpi1	7	33.90076	glucose-6-phosphate isomerase 1 [Source:MGI Symbol;Acc:MGI:95797]
NUCB1	Nucb1	7	45.13988	nucleobindin 1 [Source:MGI Symbol;Acc:MGI:97388]
LDHA	Ldha	7	46.4909	lactate dehydrogenase A [Source:MGI Symbol;Acc:MGI:96759]
ALDOA	Aldoa	7	126.3944	aldolase A, fructose-bisphosphate [Source:MGI Symbol;Acc:MGI:87994]
TALDO1	Taldo1	7	140.9721	transaldolase 1 [Source:MGI Symbol;Acc:MGI:1274789]
CTSD	Ctsd	7	141.9296	cathepsin D [Source:MGI Symbol;Acc:MGI:88562]
LAMP1	Lamp1	8	13.20916	lysosomal-associated membrane protein 1 [Source:MGI Symbol;Acc:MGI:96745]
LPL	Lpl	8	69.33314	lipoprotein lipase [Source:MGI Symbol;Acc:MGI:96820]
ATP6V1B2	Atp6v1b2	8	69.5413	ATPase, H+ transporting, lysosomal V1 subunit B2 [Source:MGI Symbol;Acc:MGI:109618]
PRDX2	Prdx2	8	85.69622	peroxiredoxin 2 [Source:MGI Symbol;Acc:MGI:109486]
COTL1	Cotl1	8	120.536	coactosin-like 1 (Dictyostelium) [Source:MGI Symbol;Acc:MGI:1919292]
HSPA8	Hspa8	9	40.71228	heat shock protein 8 [Source:MGI Symbol;Acc:MGI:105384]
H2AX	H2ax	9	44.24599	H2A.X variant histone [Source:MGI Symbol;Acc:MGI:102688]
PKM	Pkm	9	59.56365	pyruvate kinase, muscle [Source:MGI Symbol;Acc:MGI:97591]
EEF1A1	Eef1a1	9	78.38573	eukaryotic translation elongation factor 1 alpha 1 [Source:MGI Symbol;Acc:MGI:1096881]
TF	Trf	9	103.0812	transferrin [Source:MGI Symbol;Acc:MGI:98821]
PSAP	Psap	10	60.11345	prosaposin [Source:MGI Symbol;Acc:MGI:97783]
CSTB	Cstb	10	78.2615	cystatin B [Source:MGI Symbol;Acc:MGI:109514]
EEF2	Eef2	10	81.01247	eukaryotic translation elongation factor 2 [Source:MGI Symbol;Acc:MGI:95288]
UBE2N	Ube2n	10	95.35101	ubiquitin-conjugating enzyme E2N [Source:MGI Symbol;Acc:MGI:1934835]
LYZ2	Lyz2	10	117.1132	lysozyme 2 [Source:MGI Symbol;Acc:MGI:96897]
GNS	Gns	10	121.201	glucosamine (N-acetyl)-6-sulfatase [Source:MGI Symbol;Acc:MGI:1922862]
LRP1	Lrp1	10	127.374	low density lipoprotein receptor-related protein 1 [Source:MGI Symbol;Acc:MGI:96828]
PPIA	Ppia	11	6.365443	peptidylprolyl isomerase A [Source:MGI Symbol;Acc:MGI:97749]
RPS27A	Rps27a	11	29.49585	ribosomal protein S27A [Source:MGI Symbol;Acc:MGI:1925544]
IL12B	Il12b	11	44.29089	interleukin 12b [Source:MGI Symbol;Acc:MGI:96540]
PFN1	Pfn1	11	70.54268	profilin 1 [Source:MGI Symbol;Acc:MGI:97549]
PITPNA	Pitpna	11	75.47892	phosphatidylinositol transfer protein, alpha [Source:MGI Symbol;Acc:MGI:99887]
NME1	Nme1	11	93.84781	NME/NM23 nucleoside diphosphate kinase 1 [Source:MGI Symbol;Acc:MGI:97355]
VAT1	Vat1	11	101.3496	vesicle amine transport 1 [Source:MGI Symbol;Acc:MGI:1349450]
GRN	Grn	11	102.3211	granulin [Source:MGI Symbol;Acc:MGI:95832]

LGALS3BP	Lgals3bp	11	118.2836	lectin, galactoside-binding, soluble, 3 binding protein [Source:MGI Symbol;Acc:MGI:99554]
ARHGDIA	Arhgdia	11	120.4689	Rho GDP dissociation inhibitor (GDI) alpha [Source:MGI Symbol;Acc:MGI:2178103]
DLD	Dld	12	31.38128	dihydrolipoamide dehydrogenase [Source:MGI Symbol;Acc:MGI:107450]
RAB15	Rab15	12	76.84473	RAB15, member RAS oncogene family [Source:MGI Symbol;Acc:MGI:1916865]
NPC2	Npc2	12	84.80134	NPC intracellular cholesterol transporter 2 [Source:MGI Symbol;Acc:MGI:1915213]
SERPINA1F	Serpina1f	12	103.6543	serine (or cysteine) peptidase inhibitor, clade A, member 1F [Source:MGI Symbol;Acc:MGI:1915598]
CKB	Ckb	12	111.6358	creatine kinase, brain [Source:MGI Symbol;Acc:MGI:88407]
H1-3	H1f3	13	23.73744	H1.3 linker histone, cluster member [Source:MGI Symbol;Acc:MGI:107502]
H2BC1	H2bc1	13	24.11776	H2B clustered histone 1 [Source:MGI Symbol;Acc:MGI:2448375]
CTSL	Ctsl	13	64.50715	cathepsin L [Source:MGI Symbol;Acc:MGI:88564]
TKT	Tkt	14	30.27032	transketolase [Source:MGI Symbol;Acc:MGI:105992]
LGALS3	Lgals3	14	47.60521	lectin, galactose binding, soluble 3 [Source:MGI Symbol;Acc:MGI:96778]
PNP	Pnp	14	51.16854	purine-nucleoside phosphorylase [Source:MGI Symbol;Acc:MGI:97365]
CTSB	Ctsb	14	63.35991	cathepsin B [Source:MGI Symbol;Acc:MGI:88561]
PTK2B	Ptk2b	14	66.39071	PTK2 protein tyrosine kinase 2 beta [Source:MGI Symbol;Acc:MGI:104908]
DPYSL2	Dpysl2	14	67.04031	dihydropyrimidinase-like 2 [Source:MGI Symbol;Acc:MGI:1349763]
LCP1	Lcp1	14	75.36854	lymphocyte cytosolic protein 1 [Source:MGI Symbol;Acc:MGI:104808]
TPT1	Tpt1	14	76.08253	tumor protein, translationally-controlled 1 [Source:MGI Symbol;Acc:MGI:104890]
BASP1	Basp1	15	25.36336	brain abundant, membrane attached signal protein 1 [Source:MGI Symbol;Acc:MGI:1917600]
LGALS1	Lgals1	15	78.81093	lectin, galactose binding, soluble 1 [Source:MGI Symbol;Acc:MGI:96777]
ARF3	Arf3	15	98.63452	ADP-ribosylation factor 3 [Source:MGI Symbol;Acc:MGI:99432]
TUBA1B	Tuba1b	15	98.82931	tubulin, alpha 1B [Source:MGI Symbol;Acc:MGI:107804]
KRT76	Krt76	15	101.7928	keratin 76 [Source:MGI Symbol;Acc:MGI:1924305]
AHSG	Ahsg	16	22.71003	alpha-2-HS-glycoprotein [Source:MGI Symbol;Acc:MGI:107189]
SOD1	Sod1	16	90.01764	superoxide dismutase 1, soluble [Source:MGI Symbol;Acc:MGI:98351]
SOD2	Sod2	17	13.22573	superoxide dismutase 2, mitochondrial [Source:MGI Symbol;Acc:MGI:98352]
TNF	Tnf	17	35.41836	tumor necrosis factor [Source:MGI Symbol;Acc:MGI:104798]
CALM2	Calm2	17	87.74084	calmodulin 2 [Source:MGI Symbol;Acc:MGI:103250]
CSF1R	Csf1r	18	61.23367	colony stimulating factor 1 receptor [Source:MGI Symbol;Acc:MGI:1339758]
CNDP2	Cndp2	18	84.68559	CNDP dipeptidase 2 (metallopeptidase M20 family) [Source:MGI Symbol;Acc:MGI:1913304]
CFL1	Cfl1	19	5.540483	cofilin 1, non-muscle [Source:MGI Symbol;Acc:MGI:101757]
PRDX5	Prdx5	19	6.884065	peroxiredoxin 5 [Source:MGI Symbol;Acc:MGI:1859821]
PGAM1	Pgam1	19	41.90036	phosphoglycerate mutase 1 [Source:MGI Symbol;Acc:MGI:97552]
LAMP2	Lamp2		37.49023	lysosomal-associated membrane protein 2 [Source:MGI Symbol;Acc:MGI:96748]
FLNA	Flna		73.26707	filamin, alpha [Source:MGI Symbol;Acc:MGI:95556]
G6PDX	G6pdx		73.45309	glucose-6-phosphate dehydrogenase X-linked [Source:MGI Symbol;Acc:MGI:105979]
MSN	Msn		95.13965	moesin [Source:MGI Symbol;Acc:MGI:97167]
PGK1	Pgk1		105.2307	phosphoglycerate kinase 1 [Source:MGI Symbol;Acc:MGI:97555]
TMSB4X	Tmsb4x		165.9901	thymosin, beta 4, X chromosome [Source:MGI Symbol;Acc:MGI:99510]

*Chr, Chromosome

Supplement Table S4. The unique proteins from the supernatant (secretome) that presented only in Ezh2 null macrophages with LPS tolerance

Pasted	Symbol	Chr*	Position (Mbp)	Description
YWHAB	Ywhab	2	163.8369	tyrosine 3-monooxygenase/tryptophan 5-monooxygenase activation protein, beta polypeptide [Source:MGI Symbol;Acc:MGI:1891917]
LMNA	Lmna	3	88.38745	lamin A [Source:MGI Symbol;Acc:MGI:96794]
MARCKSL1	Marcksl1	4	129.4074	MARCKS-like 1 [Source:MGI Symbol;Acc:MGI:97143]
LPL	Lpl	8	69.33314	lipoprotein lipase [Source:MGI Symbol;Acc:MGI:96820]
RPS27A	Rps27a	11	29.49585	ribosomal protein S27A [Source:MGI Symbol;Acc:MGI:1925544]
PTK2B	Ptk2b	14	66.39071	PTK2 protein tyrosine kinase 2 beta [Source:MGI Symbol;Acc:MGI:104908]
AHSG	Ahsg	16	22.71003	alpha-2-HS-glycoprotein [Source:MGI Symbol;Acc:MGI:107189]
CALM2	Calm2	17	87.74084	calmodulin 2 [Source:MGI Symbol;Acc:MGI:103250]
CFL1	Cfl1	19	5.540483	cofilin 1, non-muscle [Source:MGI Symbol;Acc:MGI:101757]
LAMP2	Lamp2		37.49023	lysosomal-associated membrane protein 2 [Source:MGI Symbol;Acc:MGI:96748]

* Chr, Chromosome
